# Utilization of health belief model in comprehending diarrheal disease dynamics: a case of cryptosporidiosis in Uganda

**DOI:** 10.1186/s12889-022-14413-0

**Published:** 2022-11-02

**Authors:** Clovice Kankya, Justine Okello, Rogers Wambi, Lesley Rose Ninsiima, Methodius Tubihemukama, Christine Tricia Kulabako, Richard Asaba, James Natweta Baguma, Musso Munyeme, James Muleme

**Affiliations:** 1 Department of Biosecurity, Ecosystems and Veterinary Public Health, College of Veterinary Medicine, Animal Resources and Biosecurity, Makerere University, P.O BOX 7062, Kampala, Uganda; 2 Department of Disease Control and Environmental Health, School of Public Health, Makerere University, P.O BOX 7072, Kampala, Uganda; 3 School of Women and Gender Studies, Makerere University, P.O BOX 7062, Kampala, Uganda; 4 Department of Disease control, School of Veterinary Medicine, University of Zambia, P.O BOX 32379, Lusaka, Zambia; 5 Department of Clinical Laboratory, Mulago National Referral Hospital, P.O Box 7051, Kampala, Uganda

## Abstract

**Background:**

Diarrheal diseases contribute greatly to the reported global childhood mortality and morbidity with related social, economic consequences. This study was conducted to analyze the utilization of the Health Belief Model (HBM) theory to comprehend diarrheal disease dynamics in Uganda.

**Methods:**

Our study utilized a qualitative cross-sectional design among adult livestock farmers in selected farming communities. A total of 80 individuals were recruited and interviewed through Focus Discussion Groups (FDGs) (*n* = 6) and Key Informant Interviews (KIIs) (*n* = 8) to evaluate diarrheal disease dynamics. The scope of dynamics included but not limited to exposure risks, knowledge, and attitudes. Our results were presented using the five (5) constructs of the HBM.

**Results:**

**Perceived susceptibility;** communities believed that both humans and their animals are at high risk of different kinds of diarrheal infections. The farmers believed that majority of these diarrhea infections are hard to treat especially among animals. **Perceived severity;** farmers believed that diarrheal diseases are characterized by loss of weight, fever, emaciation, dry eyes, severe prolonged diarrhea and sudden death. **Perceived barriers;** limited knowledge and misconceptions about the diarrheal infections were great inhibitors to successful disease prevention and control. **Self-efficacy;** farmers had fear of laxity that interventions being suggested and put in place to curb diarrheal diseases such as cryptosporidiosis would wither away with time thus endemicity of the problem in the community. **Modifying factors and cues to action;** most of the farmers treat animals by themselves based on; probability, traditional knowledge and previous experience.

**Conclusion:**

Sustained public health interventional activities should therefore be undertaken by both human and animal health sectors with maximum community involvement. Communities suggested the need to increase preventive measures and promote household hygiene efforts to always wash hands with soap and running water in order to reduce the burden of diarrhea diseases such as cryptosporidiosis.

**Supplementary Information:**

The online version contains supplementary material available at 10.1186/s12889-022-14413-0.

## Introduction

Globally, diarrheal diseases continue to threaten public health with high childhood mortality and morbidity [1, 2]. According to the World Health Organization (WHO), 5.0 million children under five die annually due to diarrheal diseases [2]. The challenge is further exacerbated with the increasing zoonotic potential of the diarrheal causing pathogens including cryptosporidium species [3].

Cryptosporidiosis, a diarrheal related illness remains exceedingly high in young children with about 16% prevalence in Sub-Saharan Africa [4]. The elderly as well surfer this preventable disease due to their low immunity coupled with multiple comorbidities, and poor nutritional status [3, 5]. Cryptosporidiosis is highly fatal especially when it manifests severely and or in chronic circumstances [6]. Indeed, cryptosporidiosis is one of the ominous diarrheal diseases that affects both humans and animals yet its etiology and associated drivers remains underexplored and poorly understood [7]. Cryptosporidium parasite is an enteric pathogen that is considered the second greatest cause of diarrhea and related deaths in children after rotavirus [8–10].

It is remarkable to note that environment plays a great role in the transmission of cryptosporidium parasite because of their ability to remain viable amidst the harsh environmental conditions [11, 12]. From the zoonotic disease perspective, this diarrheal related disease is transmissible from animals to humans [10] especially within pastoral communities. In yet another dimension, communities neighboring the wild (game parks and reserves) experience constant episodes of diarrheal-like diseases [11]. Recently, in the rural communities of South Western Uganda, a study conducted by Usman et al. indicated a high prevalence of diarrheal like illness among members of communities that live proximal to game park and reserves [12]. Understanding of the drivers of such diarrheal diseases with zoonotic links requires urgent utilization of a suitable health related model such as Health Belief Model (HBM) to design appropriate strategies for better control and prevention measures.

The HBM recognizes that the health of humans, animals and environment are interlinked [13]. Therefore, its application in unearthing the diarrheal disease dynamics is very suitable for guiding health promotion and disease prevention programs in rural settings such as southwestern Uganda. At present, there are limited studies that have actualized the HBM in understanding the perceived beliefs of zoonotic disease dynamics within rural communities. This is why the current study was designed to determine the rural communities’ perceived beliefs (barriers, susceptibility, severity, benefits), self-efficacy, modified variables and cues to action on diarrheal diseases using cryptosporidiosis as a case for Uganda.

## Materials and methods

### Study design

This cross-sectional study utilized a qualitative approach in south western Uganda, Kasese district. A total of 80 individuals were interviewed using Focus Group Discussions (FGDs- *n* = 6) and Key Informant Interviews (KIIs- *n* = 8) to determine the rural communities’ perceived beliefs, self-efficacy, modified variables and cues to action on diarrheal diseases. Using a purposive sampling strategy, livestock-adult farmers were selected. The main participants for the FGDs were livestock farmers while those for the KIIs included, extension workers, husbandry officers, village health team members, heads of health centers, among other knowledgeable individuals such as herbalists.

### Study setting and population

**Fig. 1:**
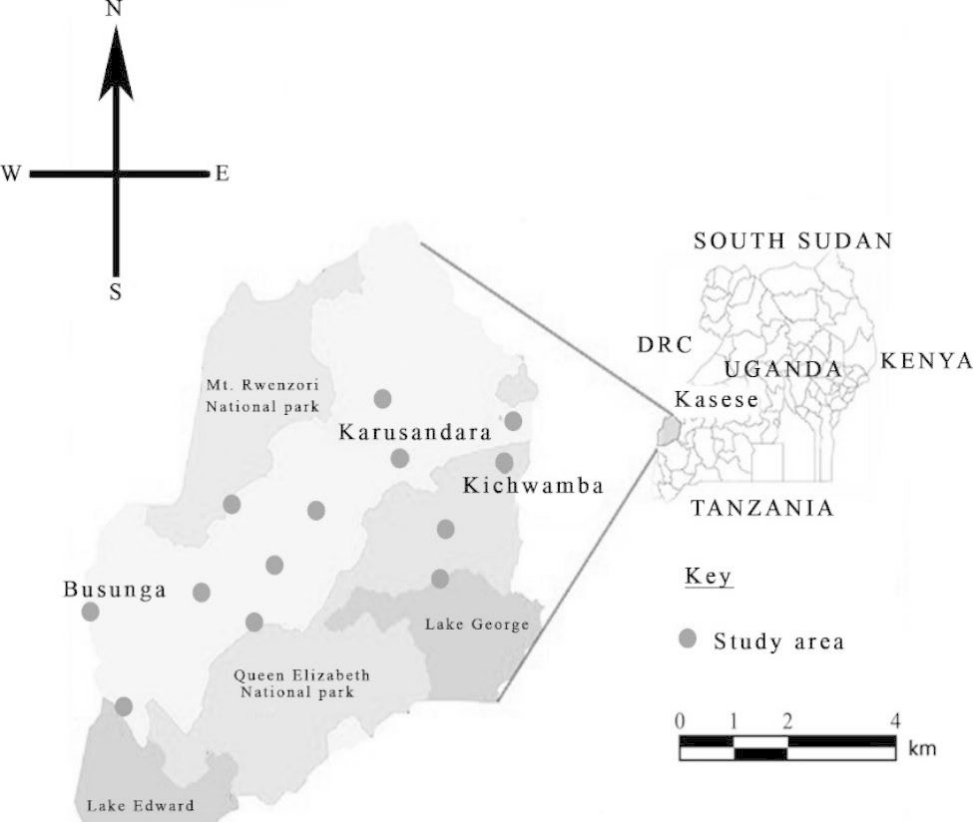
Showing a map of the study area

Kasese (Fig. 1) is located in the southwestern region of Uganda and it’s predominantly occupied by the Bakonzo, Basongora and the Bamba (UBOS, 2017). The district is also a home to many refugees from Democratic Republic of Congo (DRC). About 80% of the people in the labor force in the district are farmers. Approximately 80% of the residents of Kasese town and those along the boundaries of Queen Elizabeth National Park are mainly engaged in livestock farming and fishing respectively.

### Sampling and data collection

Livestock farmers were purposively selected in the study to ensure diversity in terms of experience and participants’ perceptions. The recruitment process was done with the help of the community health workers and local council chairperson who also acted as the “community gatekeepers” to the study site.

Data was qualitatively collected through key informant interviews and focus group discussions. FGD participants comprised of a minimum of 12 members; (6 women, 6 men). Interviews for both KIIs and FGDs were done using face to face interaction and conducted in the local languages commonly spoken in Kasese (Lhukonzo, Lusongora, Lunyoro and Bamba). The KIIs and FGDs were conducted using a topic-based interview guide that consisted of open-ended questions related to indigenous knowledge about diarrheal diseases spread, perceived beliefs (barriers, susceptibility, severity, benefits), self-efficacy, modified variables and cues to actions.

### Community identification of the disease

During the data collection process, the research team together with the livestock farmers came to a consensus that they can judge/identify diarrheal diseases caused by cryptosporidium infection. This was based on the observable signs and symptoms or health conditions for both the animals and humans by giving the local name. Cryptosporidium disease presents with prolonged watery diarrhea in animals and humans hence the local name *“okuchugura.’’*.

### Data management and analysis

To guarantee data quality and management, the moderator and note taker ensured functionality and reliability of recording devices before interviews and safety of recordings. All audio recordings were cross-checked after interviews to ascertain status and later the interviews were transcribed into verbatim and analyzed using qualitative content analysis. Notes were also taken alongside recorder by the note-taker following the interviews.

Audio-tapped data from KIIs and FGDs was first transcribed in English. The transcription process was quality controlled by well-trained qualitative data analysts and completeness check was done to ensure accuracy by the third qualitative data analysis expert. The data analysts employed the systematic procedure of collating data collected from all interviews as guided by the Graneheim and Lundman framework [14].

The research team then undertook a painstaking review process where each transcript was read thoroughly to identify key statements that were repeated several times in order to identify the codes. This process was done by two independent reviewers and the results were compared for triangulation by the third reviewer. Using NVivo (Version 12.0) pro, codes were developed from the identified topical sentences and aligned to the HBM constructs. This guided the development of sub-themes which were later merged to form a total of 6 themes guided by the HBM as stipulated in the results section of this paper.

## Results

### Perceived severity

Livestock farming communities in Kasese district reported experiences with cryptosporidium infection (commonly referred to as prolonged untreatable diarrhea) among susceptible human and animal populations. Participants from the FGDs noted that the disease was more rampant and severe among the small ruminants such as goats and sheep. They noted, that this rare disease was characterized by loss of weight, fever, emaciation, dry eyes, severe prolonged diarrhea and sudden death. The livestock keeping communities added that unlike other diarrheal diseases, this particular one “cryptosporidium infection” is hard to treat with the available veterinary regimens. This therefore ultimately culminates into livestock mortality thus affecting farmers’ livelihoods.

*“When the animals are faced with such diarrhea, we give one drug and if it refuses, we try another drug but when all these fails to work, we call the veterinary doctor (musawo) as the last hope. If they also fail in most cases, we just sit and wait for the animal to die or we sell them off cheaply.”* (FGD near national park).

*“In this community we have lost more goats than any other animal and for sure when a goat starts having diarrhea there is minimal or no hope for its recovery. Some of us just plann to sell them off.”* (FGD near Democratic Republic of Congo boarder).

The livestock farmers expressed fear that the disease apart from attacking their animals can also affect their health.

“*I remember, in one of the community health meetings carried out by Makerere University, the doctors told us that some diseases can come from animals and affect us.”* (FGD at the district health quarters)

They noted that most of these diarrheal diseases greatly impact the infants and those with chronic illness such as diabetes, Human Immunodeficiency Virus (HIV), high blood pressure among others. Some societies however associated such prolonged diarrhea and emaciation in humans to witchcraft and HIV leading to stigmatization.

“*In fact, musawo one of my neighbors got diarrhea for a week but one night as we were in the house, we had witchdoctors performing rituals at their home. They thought that perhaps this was due to evil spirits in the stomach of the affected person*” (KII near lake regions).

*“Here at the health facility, we usually receive several diarrheal-like cases and we always treat them normally but some take longer to heal. So, as the in charge, I am still confused because this seems to be more than a diarrheal disease and maybe I think it could be the one that you are telling me about.” (*KII-Health in-charge HCII*).*

### Perceived susceptibility

Livestock farmers through most of FGDs believed that both humans and animals are highly susceptible to infection by the cryptosporidium pathogen. They stated that each one of them has ever experienced a prolonged diarrhea episode even though they could not tell the actual cause. These farmers added that the problem is more prominent in households which practice free range livestock farming.

In this study, livestock farmers and their animals realized that they could both easily acquire diarrheal like infections especially if prevention and control measures are not closely observed. However, these farmers also perceived that compared to them, their children were at higher risk of acquiring the infections and suffering its tragic effects. Furthermore, the participants in the present study perceived that households that kept animals were more prone to diarrheal infections.

*“For the fact, we are also aware that regardless of age difference, both our animals and ourselves can suffer very immensely from diarrheal-like illnesses and its effects”*. (FGD near Lake Nyamunuka)

Interestingly, these farmers perceived that those livestock that were kept proximal to the game park were more at risk of acquiring the infection.

*“Our relatives who live close to the game park have animals that are always interacting freely with other wild animals. On several occasions whenever we go visiting, we find their livestock suffering diarrheal-like infections”.* (FGD near Lake Nyamunuka)

In addition, they stated that diarrheal like illnesses in both animals and humans are often pronounced during periods of intense rains.

*“The truth is during intense rainy seasons; we see high level of diarrheal like infections in both our animals and children.”* (FGD near national park).

### Perceived barriers

Livestock farming communities in Kasese district identified a number of barriers which they felt made it harder to prevent cryptosporidium infections from affecting them and their livestock. For instance, several respondents during the FGDs reported that they had limited knowledge about the infection as well as misconceptions about the disease. Only a few knew what cryptosporidium infection was after probing and description of the signs and symptoms of the disease in both humans and animals. Furthermore, findings from the 5/6 FGDs and 6/8 KIIs revealed a general misconception and myths about cryptosporidium infection. It was believed that cryptosporidium infection is misconceived and misdiagnosed as Trypanosomiasis locally known as “*ekipumpuru*” by both local farming communities and health professionals.

*“For us here in Busunga, we have never heard about that cryptosporidiosis disease but we do see the signs like severe diarrhea among our cows and children as a result of worms and sometimes due to Trypanosomiasis, and this is more common among the calves. Sometimes our cows develop wounds in the mouth (kahunu)”.* (FGD near *Busunga* Sub- County headquarters).

*“When a patient present with diarrhea, we just take it as cholera and we treat them as cholera patients but they take long to heal and some do not actually recover. Unfortunately, we cannot trace the cause of this prolonged diarrhea and may be that is why we are failing to treat our people better.” (*KII with the in-charge HC II)

It is important to note that there was a general lack of knowledge on prevention, treatment and control of cryptosporidium infection. This was attributed to the limited number of veterinarians, inefficiency of animal health workers, poor diagnosis, limited access to drug shops and self-treatment. The farmers reported that their veterinary doctor does not call them for seminars, and only become available after they are facilitated by a farmer to come and treat their livestock.

*“We are facing a very big challenge as livestock farmers; we were not informed about this disease so we completely know nothing about how it spreads and how we can control it.”* (FGD with men in Busunga)

*“Sometimes we do treat our animals with tylocine but we don’t know the exact dose that we are supposed to give our animals so we find that the medicine does not work.”* (KII in Karusandara).

*“We have ever treated our animals but they don’t get well after giving it all that medicine it finally dies. Sometimes, it can be eating and you see it losing weight and we think that it has eaten a polyethene paper.” (*FGD with men in Karusandara)

Almost all of the livestock farmers during the FGDs were concerned that the veterinary doctors were asking for a lot of money for consultation, treatment and buying medicines and they did not have that money. However, one of the animal health workers during a KII acknowledged the fact that some farmers prefer self-medication for their animals as they do not even consult before administering the medicines that they have bought from the drug shops.

*“The other challenge is that our veterinary doctors don’t make follow ups and this is because we don’t have money to facilitate them. They also delay where by you can call one in the morning but he tells you that is coming and a person spends like two days without coming, our animals then die.”* (FGD with women in Busunga)

*Worse still, some farmers just go to the clinic with their own prescription of medicine and you may find that a person ends up buying wrong medicine.* (KII with animal health worker in Busunga)

### Self-efficacy

It was reported that some individuals boiled drinking water while others did not and in addition, others used water guard (chlorine tablets) for treating their drinking water. However, some respondents reported that they are not used to drinking boiled water so they did not see a reason for starting to boil.

*“We boil the drinking water while others use water guard which they do get from nearby health centers. Some other people deliberately don’t boil their drinking water just because that they are not used to it.” “Those that drink un boiled water at times suffer from diarrheal diseases” (*FGD near National Park*).*

It was noted that livestock farmers believed that cryptosporidiosis is a serious threat to the community that needs timely interventions and coordinated efforts to prevent and control their spread. However, given the social beliefs, economic constraints and experience of some livestock farmers, there is fear of laxity that interventions being suggested and put in place to curb the disease would wither away with time thus endemicity of the problem in the community.

There was also a fear among livestock farmers that if they are not accorded with the necessary services and support from government and non-governmental organizations (NGOs) such as recruitment of veterinary doctors, free vaccination campaigns against cryptosporidium, spraying of tsetse flies among others would make prevention and control of cryptosporidiosis a greater challenge to the community. The community believed that they, alone do not have the capacity to prevent and control infections caused by cryptosporidium pathogen.

*“Thank you for sharing with us knowledge about this strange disease in our area, we would want to fight the disease in this area but we do not have the necessary capacity to sustain the suggested interventions on our own. Maybe if the NGOs and government support us we can wipe it out of the community” (KII in Karusandara).*

### Perceived benefits

Livestock farmers further felt that eradicating the germs that causes this diarrheal like infections would greatly improve the quality and quantity of their productivity. They added, that there is a strong need to provide training to farmers and extension workers regarding this rare disease that affects their health and livestock productivity.

*“When our animals suffer from diarrhea we lose a lot because their milk and meat production drastically reduces in both quality and quantity. Devastatingly, when our children are affected with diarrhea, we feel so stressed, we lose a lot of money on treatment of the disease and as well we lose a lot of time that we could have spent on our farms. Therefore, if we could be trained on what we can do to better avoid this infection, we and our animals will be healthy and have improvements in our productivity”* (FGD near Lake Nyamunuka)

### Modifying factors and cues to action

Several factors were pointed out indicating the reasons as to why farmers always do treatment of their livestock by themselves without support from a veterinarian or an extension agent. It was evident that most of the farmers treat animals by themselves and many end up treating by chance and previous experience.

*“For us we do vaccinate our cows every after three months and we do treat them with trodax for the calves and then we add berenil due to the persistence of the disease.” (*KII- near Lake Nyamunuka).

From this study, it was found out that local and traditional knowledge coupled experience greatly influences the actions taken by farmers in the management of the disease cases among their livestock.

*“We might have seen it but we don’t know the exact disease where by our cows can have diarrhea, lose weight but do not vomit so we try to give first aid like beneral, oxine, and other medicine but they finally die and sometimes when our cows have diarrhea sometimes we think that may be the cow has eaten a polythene paper when it shows up such signs”* (FGD near the district Headquarters).

In addition, livestock farmers usually treat their animals as soon as they notice any signs of diarrhea in their animals. When humans present with prolonged diarrhea, they also purchase drugs from available drug shops while others used local herbs before seeking medical attention.

*“For us we first go to the drug shops and buy tablets like flagyl, paracetamol, and syrups, or else we first look for the local herbs which is akanyasagama”* (FGD near sub county quarters)

*“Immediately after identifying that the cow is sick we go and look for medicine sometimes, we do inject berenil and Novidium, pensline, calvason and bipacon. However, some time we first, inject oxine as a first aid then we can call a veterinary doctor for proper medication.” (*FGD near DRC boarder area).

Women and children within the livestock farming communities are responsible for cleaning animal houses (*See supplementary figures*). It is imperative to note that in some communities, dung has important health benefits and is of economic importance so they don’t remove any of the animal droppings. In other communities, farmers have designated places (fecal deposition sites) where all animal droppings are kept after cleaning the houses. This dung is burnt for historical beliefs with several tagged to its ability to chase away mosquitoes and tsetse flies.

*“For us we don’t remove fecal matters from the cow’s fence and sometimes after the dung accumulates and they are dry we do burn them unless there is a customer who has come to buy them and the truck is at 50,000/= and this is done twice a year and some time we leave them because during rainy season it is being erased by rain.”* (FGD near Lake Nyamunuka)

*“Some people burn the cow dung however majority of us do not because we so much treasure cows. Actually, we sell some of this dung plus that from goats to cultivators at 30,000/= per trip” (*FGD near district quarters*)*

Hygiene promotion in homes like constructing urinal places was noted as one of the ways of preventing cryptosporidium infections. In addition, construction of fences and enclosures for the animals as a way to avoid interaction with the wild animals was yet another thought way to prevent such disease occurrences.

*“Here, we usually don’t construct urinal places but as you have seen we are surrounded by bushes so we also ease ourselves there. But from what we are hearing, we need to be supported and we construct toilets and urinals as to prevent diarrheal diseases*’’ (FGD near sub head county quarters)

*“Yes, we construct fences for the cows which doesn’t need to put the roof we don’t put even the floor but for the goats some of us in our homes do build for them. It is only like 10% that build roofs with iron sheets but with no floor. The cats sleep with us in the houses and also people have been sleeping with the pigs in the houses especially the cultivators, the Bafumbira” (*FGD near head district quarters)

## Discussion

The current study revealed a high perceived susceptibility that communities had towards diarrheal infection among livestock and humans. This was aggravated by the general lack of knowledge on prevention and control of such infections. Studies have indicated that lack of knowledge among individuals immensely affects the management of the illnesses such as cryptosporidium infection [15]. Even though several studies have been done regarding the dynamics of diarrheal diseases, hypothetically guided studies like the current one that could help in understanding the barriers associated with management of such infections are still scarce. For instance, the risk factors that could have been thought to exacerbate diarrheal-like infections in this study included; proximity to the game park, age of the both the animals and the humans [16], weather, seasonality [17, 18] and adherence to the prevention and control measures [19]. This implies that public health interventions that target community-based factors to disease need to be explored more.

Our study participants portrayed a high perceived severity where by almost all the livestock farmers said that these diarrheal diseases especially those of zoonotic nature are hard to treat. They added that there were several factors ranging from poor health service delivery (humans and animals), poverty, inadequate number of human and animal health practitioners, and high costs of health services, among others that increase severity of the infections leading to detrimental effects. Several studies have highlighted the disruption of the human and animal health services delivery systems including health facilities especially in rural areas of low- and middle-income countries (LMICs) [20]. This is further driven by poverty in these communities as well as ignorance about the use of medicines and standard operating procedures followed in managing diarrheal cases [21]. Consequently, prevention and control programmes for diarrheal infections including zoonoses should be over emphasized [17].

The perceived barriers in relation to the possible behavior of taking action for preventing and controlling these diarrheal diseases were evident in the study area. It was clear that these barriers affect the possibility of taking action to prevent diarrheal diseases in humans and animals among livestock keeping communities. Despite the large public health burden posed by these diarrheal infections such as *cryptosporidiosis*, no vaccine exists for humans and animals and treatments remains inadequate [18]. The unreliable availability of human and animal medical products, inadequate healthcare infrastructure, and a shortage of trained health staff, inadequate experience with illness among others hinder successful management of these infections [22]. In Africa, drug stock-outs have been reported in public health systems, mainly due to weak procurement and supply chain management systems and inadequate financing [23]. Therefore, national priorities should be focused on the management of diarrheal infections both in the human and animal sectors.

The perceived benefits realized in this study included prevention and control diarrheal-like infection training as well as eradication of the associated disease-causing germs. This could have been due to the fact that most people in resource limited settings like in this study always prefer prevention than cure as in a long standing saying *“prevention is better than cure”* [24].

The livestock farmers periodically vaccinate, monitor disease signs and symptoms, treatment and promotion of hygiene in homes. This could perhaps be due to the triggers such as ownership and livestock being taken as a source of livelihood for some communities [25]. Therefore, a farmer would do all it takes to ensure that the animals are safe from diseases such as *cryptosporidiosis* which would rather claim lives of their animals. Even though the findings from the present study do not quantify the burden of infection, other studies clearly reveal the high burden of the cryptosporidium infection especially in low resource settings of Sub-Saharan Africa [15]. This study being qualitative in nature, the view presented could be individual based and not necessarily that of the general population. Also the magnitude of the burden under study could not be expressed in clear quantitative terms.

## Conclusion

Despite the rigorous efforts to reduce the diarrheal diseases within a community, interventions such as limiting the number of animals enclosed in the same facilities, fencing the farming areas, cleaning their houses, training and sensitization, treatment of diarrheal infection need to be promoted. This calls for cues to action that involves multi-disciplinary teams that work within close cooperation and interaction such as veterinarians, community health workers and public health operators are essential to properly control this disease. Sustained public health education activities should therefore be undertaken by both human and animal health sectors with maximum community involvement in order to increase the success of intervention outcomes [26].

## Electronic supplementary material

Below is the link to the electronic supplementary material.

Supplementary Material 1

## Data Availability

The data that supports the findings of this study is from data collection within Kasese district. The data set generated and/or analyzed during the current study are available from the corresponding author on reasonable request.

## Abbreviations

LMICs: Low to middle income countries
et al.,: And Others
DRC: Democratic Republic of Congo
UBOS: Uganda Bureau of Standards
FGDs: Focused Group Discussions
KIIs: Key informant Interviews
SBLS: School of Biotechnical, Biosecurity and Laboratory Sciences
HIV: Human immune deficiency virus
HC: Health Centre
