# Supplementary material for: Utilization of health belief model in comprehending diarrheal disease dynamics: a case of cryptosporidiosis in Uganda
Source: BMC Public Health. 2022 Nov 2;22:2008. doi: 10.1186/s12889-022-14413-0 (PMC9628109; doi:10.1186/s12889-022-14413-0)
Supplement: Supplementary file 1 — Supplementary Material 1 [file 12889_2022_14413_MOESM1_ESM.docx]

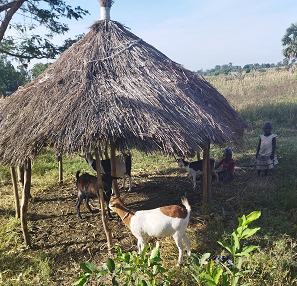


*Figure 1: showing interaction between humans, animals and the environment.*


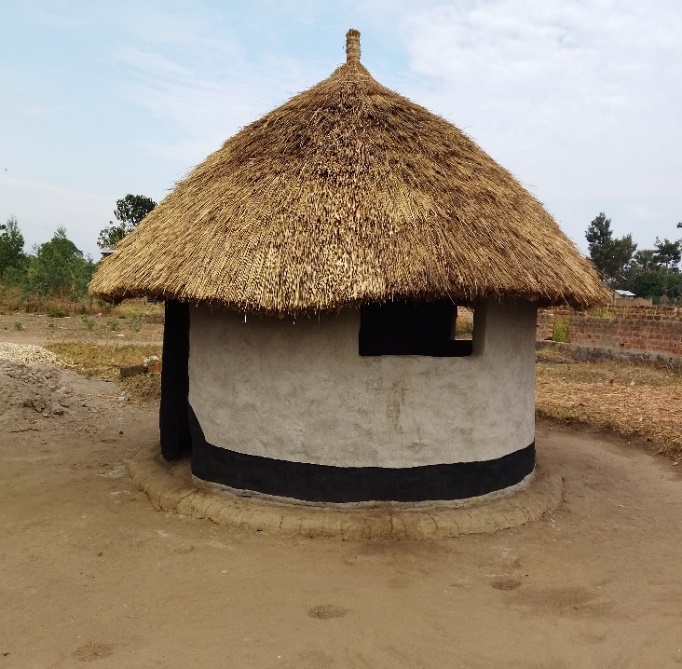


Figure 2: Showing the use of animal droppings in construction of houses.


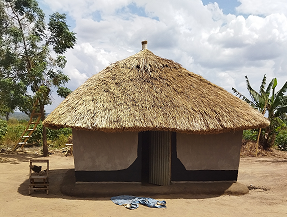


Figure 3: Showing the use of animal droppings in construction of houses.
